# Emerging Technologies for the Diagnosis of Urinary Tract Infections: Advances in Molecular Detection and Resistance Profiling

**DOI:** 10.3390/diagnostics15192469

**Published:** 2025-09-26

**Authors:** Baiken Baimakhanova, Amankeldi Sadanov, Vladimir Berezin, Gul Baimakhanova, Lyudmila Trenozhnikova, Saltanat Orasymbet, Gulnaz Seitimova, Sundetgali Kalmakhanov, Gulzakira Xetayeva, Zhanserik Shynykul, Aizat Seidakhmetova, Aknur Turgumbayeva

**Affiliations:** 1 Research and Production Center for Microbiology and Virology LLC, Bogenbay Batyr St. 105, Almaty 050010, Kazakhstan; bbbayken@mail.ru (B.B.); a.sadanov1951@gmail.com (A.S.); vberezin359@gmail.com (V.B.); bgulb@mail.ru (G.B.); barahtian@yandex.ru (L.T.); s_orazymbet@inbox.ru (S.O.); 2 Faculty of Chemistry and Chemical Technology, Al-Farabi Kazakh National University, Almaty 480012, Kazakhstan; smm170782@gmail.com; 3 Faculty of Medicine and Health Care, Al-Farabi Kazakh National University, Almaty 050040, Kazakhstan; turgumbayeva.aknur@med-kaznu.com; 4 Departament of Pediatric Infectious Diseases, S.D. Asfendiyarov Kazakh National Medical University, Almaty 050012, Kazakhstan; gulzakira.xetayeva@gmail.com; 5 Department of Emergency Medicine and Nursing, Faculty of Medicine, JSC “South Kazakhstan Medical Academy”, Al-Farabi Square 1, Shymkent 160001, Kazakhstan; aizat-seidahmetova@mail.ru

**Keywords:** urine culture, urinary tract infection, diagnostic microbiology, urobiome, molecular diagnostics, antimicrobial susceptibility testing, next-generation sequencing

## Abstract

**Background/Objectives**: Urinary tract infections (UTIs) represent a considerable challenge within the field of clinical medicine, as they are responsible for significant morbidity and intensify the operational pressures encountered by healthcare systems. Conventional diagnostic approaches, which include symptom evaluation, dipstick urinalysis, and standard urine culture, often demonstrate inadequacies in identifying atypical clinical manifestations, infections with low bacterial counts, or pathogens that show growth difficulties under typical laboratory conditions. These limitations undermine diagnostic accuracy and hinder timely therapeutic measures. **Methods**: The present manuscript is a systematic review conducted in accordance with PRISMA guidelines. A structured search was performed in PubMed, Scopus, and Google Scholar, yielding 573 records, of which 107 studies were included for qualitative synthesis. The primary aim of this systematic review is to evaluate both conventional and emerging diagnostic methods for UTIs, with specific objectives of assessing their clinical applicability, limitations, and potential to improve patient outcomes. **Results**: Recent progress in diagnostic technologies offers promising alternatives. Molecular-based assays, such as multiplex polymerase chain reaction, matrix-assisted laser desorption ionization mass spectrometry, and next-generation sequencing, have substantially improved both the precision and efficiency of pathogen identification. Furthermore, contemporary techniques for evaluating antimicrobial susceptibility, including microfluidic systems and real-time phenotypic resistance assays, enable clinicians to execute targeted therapeutic strategies with enhanced efficacy. Results of this synthesis indicate that while conventional diagnostics remain the cornerstone for uncomplicated cases, innovative molecular and phenotypic approaches demonstrate superior performance in detecting low-count bacteriuria, atypical pathogens, and resistance determinants, particularly in complicated and recurrent infections. These innovations support antimicrobial stewardship by reducing dependence on empirical antibiotic treatment and lessening the risk of resistance emergence. **Conclusions**: Nonetheless, the incorporation of these technologies into clinical practice requires careful consideration of implementation costs, standardization protocols, and the necessary training of healthcare professionals. In conclusion, this systematic review highlights that emerging molecular diagnostics and resistance-profiling tools offer substantial promise in complementing or enhancing traditional methods, but their widespread adoption will depend on robust validation, cost-effectiveness, and integration into clinical workflows.

## 1. Introduction

Urinary tract infections (UTIs) rank among the most common bacterial infections globally. Between 1990 and 2021, their incidence rose by 66.45%, reaching 4.49 billion cases with an age-standardized rate of 5531.88 per 100,000 [1]. Each year, UTIs cause approximately 400 million episodes and over 200,000 deaths, positioning them as a major driver of antimicrobial prescriptions [2]. Women are disproportionately affected, with up to 60% experiencing at least one episode during their lifetime [3], a disparity largely attributable to anatomical factors such as a shorter urethra and its proximity to the vaginal and anal regions [4]. Hormonal influences also play a role: estrogen helps sustain a *Lactobacillus*-dominant vaginal microbiome, lowering pH and protecting against uropathogen colonization [5,6]. Hormonal fluctuations during the menstrual cycle and estrogen decline after menopause diminish this protection, increasing susceptibility to recurrent UTIs [7].

In clinical practice, diagnosis is usually based on symptoms such as dysuria, frequency, urgency, suprapubic discomfort, and pyuria, with urine culture confirming the pathogen [8]. However, this approach is limited in specific populations. Infants and young children often present with nonspecific systemic symptoms (fever, vomiting, poor feeding) [9], while older adults may show confusion or appetite loss [10]. Patients with catheters or underlying neurological/urological disorders frequently exhibit persistent bacteriuria and leukocyturia, complicating differentiation between infection and colonization. Such diagnostic challenges delay treatment, promote inappropriate antibiotic use, and aggravate antimicrobial resistance (AMR) [11].

UTIs are classified by clinical and anatomical criteria. Lower UTIs include cystitis and urethritis, while upper UTIs (pyelonephritis) often involve bacteremia or urosepsis [12,13]. They are further stratified as uncomplicated or complicated. Uncomplicated UTIs (uUTIs) typically affect healthy, non-pregnant women without structural or functional abnormalities and are often managed empirically [14]. Complicated UTIs (cUTIs) arise in patients with risk factors such as obstruction, diabetes, immunosuppression, pregnancy, male sex, or indwelling devices, requiring targeted therapy due to their severity and resistance patterns [15]. Catheter-associated UTIs form a distinct subset because of their specific pathophysiology [16].

Recurrent UTIs are defined as more than two infections within six months or more than three within one year, reflecting persistence or reinfection. Chronic UTI is sometimes used for unresolved cases. Asymptomatic bacteriuria, defined as uropathogen presence at significant counts without symptoms, typically uses a ≥10^5^ CFU/mL threshold. Lower cutoffs (≥10^3^ for women with acute cystitis; ≥10^4^ for men or catheterized patients) are also clinically relevant [17]. Treatment is generally avoided, except in high-risk groups such as pregnant women, renal transplant recipients, and patients undergoing invasive urologic procedures [17,18].

**Figure 1:**
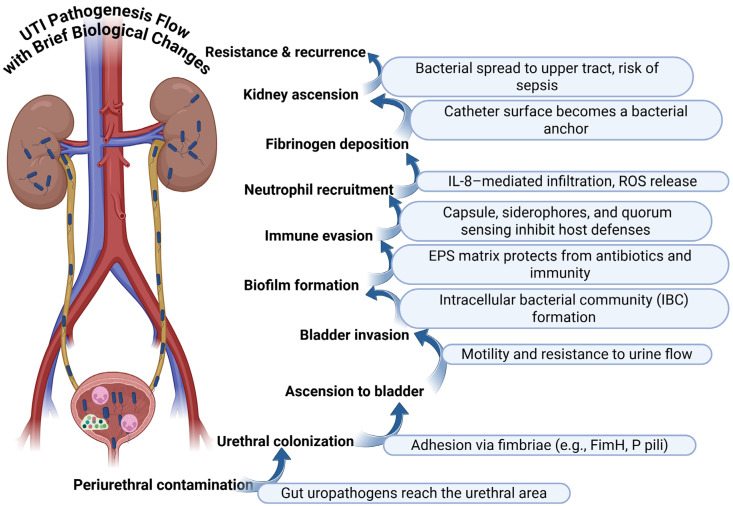
Stepwise pathogenesis of UTIs with associated biological changes [15,16,21].

In healthy individuals, urine is usually sterile or contains bacterial loads too low to cause disease [19]. Disrupted host defenses, anatomical abnormalities, or immunosuppression allow microbial invasion [20]. Uncomplicated UTIs occur in those with normal anatomy, while complicated infections are linked to structural abnormalities or catheters [16]. Gram-negative bacteria dominate, especially uropathogenic *Escherichia coli* (UPEC), which employs adhesion, biofilm formation, and immune evasion strategies to persist in the urinary tract and on catheters [15,16,21] (Figure 1). Rising AMR, particularly among multidrug-resistant Enterobacteriaceae, complicates management and underscores the need for antibiotic stewardship [22,23]. Although bacteria are the primary cause, *Candida albicans* and viruses (e.g., cytomegalovirus, human polyomavirus type 1) contribute in immunocompromised hosts, requiring precise diagnostics in these groups [24].

UTIs predominantly occur as opportunistic phenomena wherein microorganisms translocate from established reservoirs such as the gastrointestinal tract, perineum, or vagina into the urinary system [4,21]. Among the array of pathogens involved, *E. coli* persists as the principal etiological agent, accounting for approximately 65% to 75% of all documented instances globally. Despite the predominance of *E. coli* as the most prevalent pathogen, the microbial composition associated with UTIs exhibits considerable heterogeneity. In cases characterized as uncomplicated infections, *Staphylococcus saprophyticus* and *Klebsiella pneumoniae* frequently emerge as primary pathogens, indicative of their ability to capitalize on advantageous host environments [25]. Conversely, cUTIs, which manifest in conjunction with underlying health conditions such as diabetes, immunosuppression, or the presence of indwelling catheters, are generally linked to a more extensive and resistant cohort of pathogens. This cohort encompasses *Pseudomonas aeruginosa*, *Proteus mirabilis*, *Serratia marcescens*, *Enterobacter* species, and *Enterococcus* species, all of which possess the capability for sustained colonization and heightened antimicrobial resistance. Nevertheless, the clinical significance of certain microorganisms remains contingent upon specific contexts. For example, the detection of *Staphylococcus aureus* in urine may suggest a hematogenous dissemination rather than a primary urinary infection, while the identification of *Candida* species is often correlated with extended catheterization or the utilization of broad-spectrum antibiotics, thus complicating interpretation. In addition to these well-established pathogens, a variety of rare yet clinically pertinent bacteria exhibiting stringent growth requirements, alongside emergent species whose categorization as either commensal or pathogenic remains contentious, further obfuscate diagnostic interpretations [25,26,27].

In light of the complex microbial landscape, urine culture and urinalysis remain the standard diagnostic modalities for UTIs. Conventional culture requires up to 48 h for results, often necessitating empiric therapy before pathogen identification and susceptibility profiles are available [28]. This delay risks both under-treatment and inappropriate antibiotic use, further contributing to antimicrobial resistance [29]. These limitations have driven the development of novel platforms that provide faster, more sensitive, and specific results. Molecular techniques such as multiplex polymerase chain reaction (PCR), loop-mediated isothermal amplification (LAMP), and next-generation sequencing (NGS) can detect fastidious organisms and mixed infections that traditional culture may miss [30]. Advanced methods are also increasingly able to combine species identification with real-time antimicrobial susceptibility testing, narrowing the gap between presentation and targeted therapy. Their clinical adoption will depend on diagnostic precision, interpretability, cost-effectiveness, and accessibility across different healthcare environments. Moreover, the increasing acknowledgment of the urinary tract microbiome challenges the long-standing notion of urine being sterile, suggesting that host–microbe interactions may be more dynamic than previously perceived. However, sequencing-based clarification of the urinary microbiome may reveal multiple organisms, some pathogenic and others commensal or nonpathogenic, which complicates diagnostic interpretation and may initially obscure clinical decision-making. At the same time, the reevaluation of low bacterial counts should be approached with caution, as indiscriminate treatment of such findings risks therapeutic polypragmasia; therefore, clinical correlation with symptoms, host context, and inflammatory markers remains essential [18].

Despite extensive epidemiological data and established culture-based frameworks, there remains a critical gap in integrating and evaluating novel molecular and phenotypic diagnostic technologies for UTIs within clinical practice. This review critically examines contemporary and emerging approaches, emphasizing their advantages, limitations, and implications for patient outcomes and antimicrobial stewardship. Particular attention is given to nucleic acid amplification assays, NGS, and rapid susceptibility platforms, while also addressing regulatory, economic, and health system perspectives to guide judicious implementation and future research priorities.

## 2. Materials and Methods

**Figure 2:**
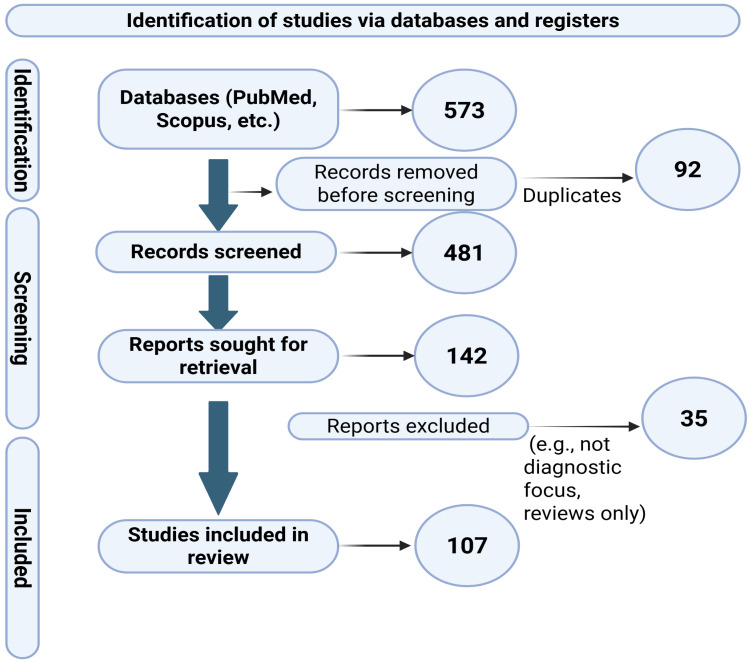
PRISMA flow diagram summarizing the literature selection process. The diagram specifies the exact number of records identified from each database, the number of duplicates removed, and the reasons for exclusion of 35 reports (e.g., *n* = 18 without diagnostic focus, *n* = 9 narrative reviews, *n* = 8 animal studies).

The review process adhered to the PRISMA (Preferred Reporting Items for Systematic Reviews and Meta-Analyses) guidelines [31]. Searches were conducted in PubMed (*n* = 210), Scopus (*n* = 190), and Google Scholar (*n* = 173), yielding a total of 573 records. Duplicates (*n* = 92) were identified using EndNote X9 (Clarivate Analytics, Philadelphia, PA, USA) automated duplicate removal complemented by manual verification. Redundant entries were expunged, and screening was executed through the appraisal of titles and abstracts. Two independent authors (G.S. and A.Sd. (Aizat Seidakhmetova)) conducted the initial title and abstract screening; full-text review was subsequently performed by the same authors. Disagreements were resolved by consensus, and when unresolved, by consultation with a third senior reviewer (L.T.). Full texts of potentially pertinent articles were subsequently evaluated for eligibility. Ultimately, a total of 99 studies were selected for qualitative synthesis. A final update search was performed in all databases in March 2024 to ensure inclusion of the most recent studies. Grey literature was not included. Non-English studies (*n* = 12) were excluded, as the review focused on English-language publications to ensure consistency of data extraction and interpretation. The study selection procedure is depicted in the PRISMA flow diagram (Figure 2).

A comprehensive literature review was executed to ascertain publications pertaining to advancements in diagnostic technologies for UTIs. Investigations were undertaken across PubMed, Scopus, and Google Scholar, utilizing a confluence of keywords and Medical Subject Headings (MeSH) terms intrinsically linked to the scope of this manuscript. Boolean operators (“AND”/“OR”) were employed in the search strings; for example, in PubMed the query was structured as: (“urinary tract infection diagnosis” OR “UTI diagnosis”) AND (“PCR” OR “NAAT” OR “LAMP” OR “sequencing” OR “molecular diagnostics”). The search methodology encompassed the ensuing terms: “urinary tract infection diagnosis,” “nucleic acid amplification tests (NAATs),” “polymerase chain reaction (PCR) UTI,” “DNA/RNA hybridization UTI,” “next-generation sequencing UTI,” “metagenomic sequencing UTI,” “rapid antimicrobial resistance detection UTI,” “molecular diagnostics for UTIs,” and “point-of-care testing UTI.”

Articles were incorporated if they concentrated on the evolution, validation, or clinical application of UTI diagnostic methodologies, encompassing molecular assays, hybridization-based technologies, sequencing platforms, or integrated antimicrobial resistance profiling. Eligible study types included original research articles, systematic reviews, meta-analyses, and narrative reviews. Exclusion criteria comprised studies limited to therapeutic interventions, investigations employing animal models, and reports extraneous to diagnostic methodology. Only publications in the English language were deemed suitable for inclusion. The 99 included studies encompassed a range of designs (original research articles, systematic reviews, and meta-analyses), diverse patient populations, and multiple diagnostic modalities, with main outcomes focused on diagnostic accuracy, clinical applicability, and antimicrobial resistance profiling.

## 3. Urinary Diagnostics in Transition: Standard Culture, Enhanced Methods, and the Urobiome

Urine culture has anchored UTI diagnosis for over seven decades, yet its clinical meaning depends on preanalytical handling, the plated volume, the atmosphere and duration of incubation, and the identity and purity of recovered colonies [32,33]. Despite guideline-driven standardization, two constraints continue to limit clinical impact: slow turnaround that delays targeted therapy, and historical semiquantitative thresholds such as 10^5^ colony-forming units per milliliter that were derived from specific cohorts and outcomes and are not universally applicable across species or patient groups [34,35]. Conventional workflows rely on short aerobic incubation, which long encouraged the view that a negative culture reflects a sterile bladder; contemporary studies indicate that nonrecovery of bacteria is chiefly a function of test conditions rather than urinary biology, underscoring the need to interpret culture results within a broader diagnostic framework [28,29].

The processes of specimen collection and management prior to plating significantly influence the insights that a culture can provide, as the methodologies employed, timing, thermal conditions, and choice of preservatives dictate both the enumeration of colonies and the composition of species [36,37,38,39]. The initial inclination towards catheter collection to mitigate contamination resulted in an increase in iatrogenic infections, prompting the adoption of midstream clean catch as the normative approach [40]. First morning voids may exhibit elevated bacterial concentrations; however, this variance rarely modifies standard collection protocols. Specimens should be analyzed expeditiously or maintained at 4 °C if delays surpass approximately thirty minutes to two hours; the application of boric acid at a concentration of 20 g/L is frequently utilized to stabilize quantitative ratios for up to forty-eight hours when prompt processing is impractical [41,42,43,44,45]. Minor deviations from these parameters can influence results across critical decision thresholds: refrigeration inhibits the proliferation of periurethral flora, yet extended storage may compromise the recovery of fastidious organisms; preservatives aid in maintaining counts, but extremely low initial burdens can still result in underdetection. In neonates, bag collection is associated with significant contamination; thus, catheter or suprapubic aspiration is favored for diagnostic sampling, whereas in adults, insufficient cleansing, vaginal discharge, menstruation, and prolonged catheter dwell times contribute to mixed growth and complicate interpretation. Recent administration of antibiotics within the preceding twenty-four to forty-eight hours can hinder recovery despite persistent symptoms, underscoring the importance of documenting both exposure and collection methodology in the laboratory report [46,47]. When preanalytical constraints are suspected, it is more informative to repeat the collection, augment the inoculated volume, or implement extended culture conditions than to regard a borderline value as conclusive; additionally, correlating culture results with objective inflammation and the clinical scenario diminishes both false negatives and false positives (Table 1).

Semi-quantitative urine culture is predicated on the inoculation of a specified volume, typically ranging from one to ten microliters, whereby the plated volume dictates the practical threshold for detection and consequently influences the probability of isolating low-abundance pathogens [42]. Upon incubation, laboratories evaluate both the quantity and purity of the growth, subsequently determining which colony types warrant identification and antimicrobial susceptibility testing, generally confining comprehensive analyses to one or two isolates in accordance with guideline frameworks that encompass collection methodologies, patient demographics, and organism classification [45,46,47]. In the context of voided specimens, the recovery of a uropathogen exceeding ten to the fifth colony-forming units per milliliter is typically interpreted as the most compelling evidence of clinical relevance, while the thresholds are generally reduced by approximately one order of magnitude for samples obtained invasively; cultures derived from meticulously collected healthy subjects frequently yield no growth [34,45]. These established conventions, while beneficial, are not without flaws. Mixed growth is often categorized as contamination, yet in specific clinical scenarios, it may indicate authentic polymicrobial disease; low counts approaching the detection threshold could signify incipient infection rather than mere background noise; and recent antibiotic administration may inhibit recovery even in the presence of persistent symptoms [42,46,47]. Furthermore, the time required for both organism recovery and antimicrobial susceptibility testing implies that results seldom inform same-day clinical decisions, thereby promoting empirical treatment and magnifying the consequences of any misclassification associated with historical quantitative thresholds that were established based on limited cohorts and subsequently extrapolated across various contexts [34,35,47,48]. Consequently, best practices necessitate the explicit documentation of collection methodology and inoculum volume, standardized annotations for mixed growth, and reflexive protocols that prompt repeat collection or enhanced culture in instances where laboratory results are at odds with pretest probabilities.

The application of a singular quantitative threshold across all taxa and patient cohorts excessively simplifies the multifaceted infection dynamics and leads to overlooked or misinterpreted pathology, as certain symptomatic infections manifest at concentrations approximating ten to the second to ten to the third colony-forming units per milliliter, where contamination and genuine infection intersect and where conventional inocula near their detection thresholds [49]. Analysis must consider the host context because pediatric subjects, pregnant individuals, catheterized or instrumented patients, those with neurogenic bladder, and immunocompromised hosts exhibit varying risks of contamination, distinct baseline microbiota, and differing probabilities of polymicrobial infection; culture alone is insufficiently reliable to differentiate colonization from active disease in numerous of these contexts [46,47,48]. Traditionally, nephrology and urology literature has regarded most polymicrobial findings as contamination, on the premise that a true pathogen inhibits the growth of other species. However, emerging molecular diagnostics increasingly reveal genuine polymicrobial infections, particularly in catheter-associated and immunocompromised populations. This discrepancy underscores the importance of careful contextual interpretation: while many polymicrobial results may indeed represent contamination, in selected clinical settings, they may reflect clinically relevant coinfections. Consequently, clinical integration is imperative: correlate quantitative findings with indicators of inflammation, symptom intensity, and progression, and the methodology of specimen collection; account for recent antibiotic administration and device utilization; and escalate beyond standard culture methodologies when a high clinical probability coincides with negative or borderline results [34,42,50,51]. To facilitate consistent decision-making at both the laboratory and clinical settings, scenario-specific thresholds and action protocols ought to be implemented rather than a singular universal cutoff (Table 2).

Enhanced quantitative urine culture (EQUC) amplifies diagnostic yield by employing larger inocula, supplementary media, alternative incubation environments such as microaerobic, anaerobic, or carbon dioxide-enhanced conditions, and prolonged incubation durations that permit the emergence of slow-growing and fastidious organisms [54,55]. These modifications diminish the practical detection threshold in comparison to one microliter aerobic protocols and reveal taxa that conventional workflows overlook, encompassing underrecognized uropathogens such as *Aerococcus urinae*, *Actinotignum schaalii*, *Alloscardovia omnicolens*, lipophilic *Corynebacterium*, including *Corynebacterium urealyticum*, *Gardnerella vaginalis*, and *Bordetella hinzii* [51,52,53,54]. EQUC is particularly elucidative when clinical probability is elevated yet routine culture is negative or indicates low counts near the detection threshold, and when mixed growth could signify genuine polymicrobial disease rather than contamination. This methodology augments organism recovery; it also necessitates meticulous clinical integration to circumvent the misinterpretation of colonization in low-risk contexts [46,47,51,52,53].

Molecular profiling of urinary communities, predominantly utilizing 16S rRNA gene sequencing, identifies many of the same populations that EQUC elucidates and broadens detection to organisms that are challenging to culture, thereby underscoring that negative growth under standard conditions reflects test parameters rather than intrinsic sterility [55,56]. These methodologies frequently discern commensals and low-abundance organisms in both symptomatic and asymptomatic individuals, which complicates interpretation and bolsters a community-based perspective in which host factors and population structure modulate symptoms [57]. Initiatives to standardize urinary microbiome workflows and reporting have been proposed; however, widely accepted clinical standards and actionable interpretive frameworks remain limited, and critical inquiries persist regarding the application of these data for individual patient management.

A practical stewardship framework associates method selection with pretest probability and sample quality. Standard semi-quantitative culture is suitable for uncomplicated presentations with moderate probability, while discrepancies between symptoms and a negative or borderline result should instigate reflex actions such as repeat collection with guided technique, increased inoculated volume, and EQUC with extended atmospheres and media. Metagenomic testing is reserved for specific scenarios that encompass recurrent symptoms with prior negative cultures, antecedent antibiotics that may inhibit growth, suspected fastidious organisms, and complex hosts such as those with devices or neurogenic bladder, acknowledging that results must be evaluated against pyuria, collection methodology, organism purity, and the progression of symptoms [34,42,51,52,53,55].

Turnaround duration constrains immediate decision-making because organism recuperation and phenotypic antimicrobial susceptibility evaluation necessitate sequential incubations, which influences initial empirical therapy and magnifies the repercussions of any misclassification attributable to historical quantitative thresholds [34,42]. As results are obtained, clinicians should either de-escalate or escalate therapy by incorporating organism identity, purity of growth, quantitative burden, host context, and objective inflammation, whilst recognizing that low counts near the detection threshold can signify early infection in the appropriate clinical context [42,50,51]. Expedited phenotypic assays and targeted genetic markers of resistance can narrow options while awaiting comprehensive susceptibility reports, yet they complement rather than supplant culture-based susceptibility testing [34,36].

The introduction of EQUC and metagenomic assays necessitates investment in media and incubator capacity, atmospheric control, personnel training, and modifications to the laboratory information system such that collection methodology, inoculated volume, storage conditions, and reflex protocols are documented and reported consistently [41,42,43,44,46,47,51,52,53]. Quality programs ought to monitor contamination and mixed growth rates by collection methodology, frequency of repeated collections, time to actionable results, concordance between methodologies, and downstream effects on antimicrobial utilization and patient revisits. Clear reporting templates that delineate preanalytical conditions and furnish standardized interpretive comments diminish variability among clinicians. Expenses escalate with prolonged culture and sequencing; thus, laboratories should confine advanced methodologies to circumstances where management is likely to alter, and health systems should assess the equilibrium between elevated test costs and potential savings derived from reduced returns, diminished unnecessary antibiotic prescriptions, and enhanced targeting of therapy [51,52,53].

Priority investigations should correlate method selection to patient-centered outcomes such as symptom resolution, recurrence, adverse events, and antibiotic exposure, with predefined strata for host groups and organism classes [16,48]. Consensus reporting standards are requisite for EQUC and urinary microbiome assays, encompassing quantitative conventions, organism lists categorized by clinical significance, and terminology for uncertainty [53]. Trials should evaluate reflex algorithms that delineate when to repeat collections, when to augment inoculum, when to implement EQUC, and when to contemplate molecular profiling, and should encompass cost-effectiveness analyses that integrate laboratory resources, time to appropriate therapy, and healthcare utilization [55]. Ultimately, research should address how to differentiate colonization from infection across species at low counts, how recent antibiotic exposure and device biofilms influence detection, and how microbiome characteristics can be transmuted into actionable risk stratification without resultant overdiagnosis [51,52,53].

## 4. Urinary Diagnostics: Improved Bacteriuria Screens, Multiplex Nucleic Acids, Sequencing, and Phenotypic Methods

Expeditious, direct-from-specimen diagnostics are being formulated to diminish the time to outcome and to mitigate unnecessary cultures by triaging negative specimens at an early stage. Current methodologies encompass enhanced bacteriuria screening, multiplex nucleic acid amplification assays, next-generation sequencing of urinary microbiota, and novel phenotypic strategies for antimicrobial susceptibility assessment (Table 3). The primary objectives are to achieve earlier, more dependable exclusion of infection, swifter organism identification, and accelerated guidance for therapeutic interventions, while ensuring that results remain interpretable within clinical context and laboratory workflows.

Screening instruments endeavor to maintain the negative predictive value of urinalysis while diminishing detection thresholds and expediting subsequent procedures (Table 4). The sole innovative Food and Drug Administration (FDA)-sanctioned rapid bacteriuria assay is BacterioScan 216Dx (BacterioScan, Inc., St. Louis, MO, USA), a light-scattering technique reporting in approximately three hours with sensitivity of 96.5 percent and specificity of 72 percent compared to culture [58,59]. Beyond the borders of the United States, Alifax Uro-Quick (Alifax S.r.l., Padua, Italy) provides analogous three-hour detection and has been employed for over a decade [60]. Both systems triage specimens for same-day matrix-assisted laser desorption ionization time-of-flight mass spectrometry by indicating adequate biomass [58,61]. Usense Jimini (Module Innovations Pvt. Ltd., Pune, India) is devoid of labels; gold electrodes capture interleukin-8, prostaglandin E2, and lipopolysaccharide, yielding results within five minutes; rapid susceptibility applications are currently under scrutiny [62]. Catalase-based Uriscreen (Savyon Diagnostics Ltd., Ashdod, Israel) exhibited modest performance relative to culture in obstetrics and pediatrics (sensitivity 60.7 and 67 percent; specificity 89.3 and 69 percent) [63,64]. UTRIPlex (Global Access Diagnostics, Thurleigh, Bedfordshire, United Kingdom), assessing three urinary inflammatory markers, demonstrated sensitivity of 21 percent and specificity of 94 percent within the same pediatric cohort [64]. UTI-lizer (Quotient Diagnostics Ltd., Camberley, United Kingdom), a “digital dipstick,” employs microreactors incubated for approximately eight hours and displays a high negative predictive value exceeding 97 percent, but exhibits considerable variability in positive predictive value, ranging from 0 to 90 percent. Pattern’s single-cell droplet imaging (Pattern Biosciences, Austin, Texas, USA) reports negative predictive value approaching 98 percent and Gram classification; development persists toward direct identification and rapid susceptibility [65,66].

Panels that enhance microbial nucleic acids directly from urine offer the potential for organism identification and the detection of selected resistance determinants within a matter of hours as opposed to days. Their merits encompass rapidity, analytical sensitivity, and the capacity to identify multiple targets concurrently, including organisms that are challenging to culture. Their constraints involve the detection of nucleic acids from nonviable organisms following recent antibiotic administration, incomplete coverage of all conceivable species and resistance mechanisms, and the necessity for meticulous interpretation when inflammation is absent or when quantitative signals are minimal. Optimal applications include patients with a substantial clinical likelihood and previous negative cultures, individuals recently subjected to antibiotics, and contexts where early organism-directed therapy could alter clinical outcomes. Results should be correlated with culture or enhanced culture for phenotypic susceptibility, and documented with standardized remarks that elucidate how targets were selected and what was not examined. The clinical utility of rapid diagnostics is particularly pronounced in young infants presenting with fever but no other symptoms, where timely confirmation of UTI can preclude the need for invasive procedures such as lumbar puncture and cerebrospinal fluid analysis under anesthesia, thereby supporting safer and more efficient patient management.

Next-generation sequencing (NGS), encompassing 16S ribosomal RNA gene sequencing, characterizes the urinary microbiome and frequently uncovers numerous organisms that extended culture recovers, in addition to taxa that are challenging to cultivate under standard conditions. This sensitivity reconceptualizes a negative growth result as a consequence of testing conditions rather than evidence of sterility; however, it simultaneously presents interpretive difficulties due to the presence of commensals and low-abundance organisms in both symptomatic and asymptomatic individuals [63]. Practical implementation necessitates transparent reporting of community composition, quantitative or semi-quantitative signals, and explicit directives on how to amalgamate findings with symptoms, inflammation, and collection methodologies. Until standardized workflows and outcome-linked interpretive frameworks are broadly embraced, sequencing is optimally suited for complex or recurrent presentations, for resolving discordant culture results, and for targeted discovery rather than routine frontline testing [64,65].

Phenotypic methodologies that function directly on urine aspire to reduce the duration required to transition from empirical therapy to targeted therapy [34]. Approaches currently under development include single-cell compartmentalization with imaging of growth and metabolic activity, optical or electrochemical readouts of drug response, and miniaturized culture systems that quantify growth in hours instead of days. Preliminary data from the aforementioned single-cell platform demonstrate a high negative predictive value for the detection of bacteriuria and encouraging organism classification, with ongoing efforts to extend the methodology to rapid susceptibility testing for urinary pathogens [64]. As these instruments advance, laboratories will necessitate explicit criteria for when a rapid phenotypic result is independently actionable and when corroboration by standard culture and susceptibility testing remains essential, alongside validation studies that associate earlier results with enhanced patient outcomes.

## 5. Advances in Molecular Diagnostics for Urinary Tract Infections: Amplification, Hybridization, and Sequencing Approaches

Emerging nucleic acid-based diagnostic platforms are increasingly being formulated to augment the detection and characterization of UTIs (Table 5). Traditional culture-based methodologies, although regarded as the gold standard, are impeded by protracted turnaround times, constrained sensitivity for fastidious organisms, and challenges in mixed infections. These constraints have expedited the advancement of molecular platforms capable of swiftly identifying multiple pathogens, detecting antimicrobial resistance (AMR) genes, and providing clinically actionable information within hours rather than days. By categorizing the available assays into classifications such as amplification, hybridization, and next-generation sequencing, this overview underscores not only the technical diversity but also the evolving diagnostic paradigm. Hence, the information elucidates how molecular innovation is progressively transforming routine UTI management.

NAATs epitomize the most thoroughly advanced category of molecular diagnostics for UTIs, providing expeditious and exceptionally sensitive pathogen identification. In contrast to traditional culture techniques, NAATs possess the ability to discern organisms at minimal colony-forming unit thresholds, occasionally as low as 10^2^–10^3^ CFU/mL, thereby facilitating prompt diagnosis and intervention. However, the clinical significance of such low-level detections remains context dependent, and interpretation requires correlation with symptoms, inflammatory markers, and host status to avoid overtreatment. Multiplex PCR platforms augment this potential by simultaneously detecting multiple pathogens and antimicrobial resistance (AMR) markers, which is particularly advantageous in intricate UTIs where polymicrobial infections are prevalent. Loop-mediated isothermal amplification (LAMP) methodologies further improve accessibility by obviating the necessity for intricate thermal cycling, rendering them potentially suitable for resource-limited environments [86,87,88,89]. Nonetheless, NAATs are not devoid of challenges. False positives may arise from the identification of nonviable organisms, while the incapacity to quantify bacterial load may obfuscate clinical interpretation. Furthermore, the expense of consumables and specialized apparatus persists as an impediment to widespread adoption beyond centralized laboratories. Despite these constraints, NAATs bridge the significant chasm between symptom onset and definitive therapy by abbreviating diagnostic delays from days to mere hours [90,91]. Consequently, they signify a substantial advancement toward personalized and precision-based management of UTIs, particularly in populations experiencing recurrent or resistant infections.

Most multiplex assays encompass AMR targets such as *mecA*, *vanA/vanB*, and Gram-negative β-lactamases, including *blaCTX-M* and carbapenemases (*blaKPC*, *blaNDM*, *blaVIM*, *blaIMP*, *blaOXA-48*). These indicators are well-established for bloodstream infections but remain inadequately researched in urine specimens [86]. In practice, *S. aureus* is an infrequent etiological agent of uncomplicated UTIs. Conventional therapy involves doxycycline or trimethoprim, with elevated susceptibility rates even among methicillin-resistant *S. aureus* (MRSA). Nevertheless, the urinary detection of S. aureus may indicate disseminated infection or a complicated UTI, wherein *mecA* becomes pertinent to inform vancomycin administration [87]. For vancomycin-resistant *Enterococcus* (VRE), nitrofurantoin and doxycycline frequently preserve effectiveness, and aminopenicillins may remain clinically efficacious for uncomplicated UTI despite minimum inhibitory concentrations (MICs) surpassing the susceptible breakpoint. Consequently, *vanA/vanB* status may confer limited utility in that context [88,89]. Among Gram-negatives, *CTX-M* information may be superfluous for uncomplicated UTI but can elucidate therapy for cUTI and pyelonephritis [90]. Several UTI-centric panels under development incorporate markers aligned with prevalent UTI pharmacotherapeutics, for instance, *sul1* for sulfonamides and *qnrB/qnrS* for fluoroquinolones. Resistance forecasting from a restricted gene set is ambiguous and likely region-dependent, as trimethoprim-sulfamethoxazole resistance may reflect *dfrA*, and fluoroquinolone resistance is often driven by chromosomal gyrase mutations rather than plasmid-mediated *qnr* genes [91]. Moreover, genotype does not invariably predict phenotype. Expression levels, porin alterations, efflux mechanisms, inoculum effects, and mixed populations can modify observed susceptibility in vivo [86]. Results should be interpreted with local antibiograms and clinical severity considered, and confirmatory phenotypic antimicrobial susceptibility testing remains crucial when treatment decisions entail substantial risk.

Point-of-care NAAT technologies for urinary tract infection are progressing from experimental prototypes to pragmatic clinical instruments, aiming to provide organism identification and selected resistance markers at or proximal to the bedside while maintaining acceptable analytical rigor and clinical interpretability. Consequently, platforms now prioritize expedited workflows, streamlined sample processing, and standardized outputs that clinicians can utilize within a singular visit. The Lodestar Dx UTI LAMP assay (Llusern Scientific Ltd., Cardiff, UK) targets six bacterial species with a projected limit of detection of 10^4^ colony-forming units per milliliter in approximately 0.5 h, the CLoNET assay (Fraunhofer Institute for Interfacial Engineering and Biotechnology IGB, Stuttgart, Germany) concentrates on *E. coli*, and the Vivalytic UTI research-use-only panel (Randox Laboratories Ltd., Crumlin, United Kingdom, in partnership with Bosch Healthcare Solutions GmbH, Waiblingen, Germany) interrogates approximately twenty bacterial targets plus eight resistance genes in under three hours. Furthermore, a meta-analysis of polymerase chain reaction applied to urine reported a pooled sensitivity of 80.8% (73.4–86.6%) and specificity of 83.7% (52.7–95.9%) in comparison with standard culture, with an estimated 16% false-positive rate; although these data do not derive from the most recent commercial systems, they accentuate persistent concerns regarding specificity [68]. Moreover, this risk is exacerbated in populations with a high prevalence of asymptomatic bacteriuria, such as residents in post-acute and long-term care environments, where up to 50% of antimicrobial prescriptions are unwarranted; introducing NAAT as a primary test may inadvertently amplify overtreatment [69]. Accordingly, we concur that NAATs should not be applied indiscriminately in such contexts. Their use must be coupled with strict stewardship frameworks and clinical correlation, ensuring that positive results in the absence of symptoms do not automatically prompt therapy. This approach mitigates the risk of therapeutic excess while preserving the advantages of rapid molecular detection. In response, numerous position statements counsel against routine NAAT utilization in these contexts and advocate for culture-based testing as the default methodology [70,71].

Because analytical sensitivity alone does not ensure clinical applicability, reporting strategies should intentionally prioritize clinical actionability. Therefore, laboratories can elevate reporting thresholds by issuing results only when signal intensities correspond to ≥10^4^ or ≥10^5^ CFU/mL, or alternatively, provide semi-quantitative tiered outputs that contextualize organism load [92]. In addition, several stewardship safeguards can further mitigate harm: correlate NAAT results with urinalysis or pyuria criteria prior to reporting; suppress low-abundance detections that lack concordant symptoms or inflammatory markers; append standardized interpretive comments that differentiate contamination, colonization, and infection; and integrate decision-support prompts within the electronic health record that encourage vigilant observation or deferred prescriptions when appropriate. Where practicable, platforms should also exhibit cycle-threshold or copy-number bands mapped to prespecified action categories, which assist clinicians in translating molecular signals into bedside decisions.

The CAPTURE UTI platform is a hybridization-based assay that targets unamplified RNA and is conceived to yield results in approximately two hours while processing numerous specimens concurrently, and because it quantifies transcripts rather than genomic DNA, it preferentially reflects viable, metabolically active organisms and can mitigate background from residual nonviable nucleic acid. Moreover, transcript-directed detection facilitates panel designs that encompass expression signatures of crucial virulence factors and resistance determinants, and it generates an opportunity to amalgamate microbial signals with host-response transcripts to enhance the discrimination of infection from colonization in symptomatic patients. Nevertheless, this methodology introduces distinct pre-analytic and analytic challenges, including the lability of RNA in urine, the necessity for immediate stabilization and RNase control, rigorous internal spike-in controls and normalization strategies, and meticulously defined limits of detection that are typically superior to multiplex polymerase chain reaction, which constricts the detectable repertoire and may overlook early infections or asymptomatic bacteriuria. Given the urinary-tract-specific utility concerns observed for blood-culture-oriented resistance targets such as *mecA*, *vanA/vanB*, and *blaCTX-M* [85,86,87,88,89,90], panel content should underscore markers aligned with first-line urinary tract infection therapies, for instance sul1 and *qnrB/qnrS*, while acknowledging that predictive accuracy for these classes is contingent on local mechanisms and may be compromised by chromosomal mutations or non-genetic resistance pathways [91]. In order to safeguard clinical specificity, laboratories can adopt semi-quantitative reporting thresholds that reflect culture action points, for example, issuing positive results only when signal intensity corresponds to ≥10^4^–10^5^ colony-forming units per milliliter, and they can incorporate reflex algorithms that pair CAPTURE results with urinalysis or pyuria criteria, stewardship messaging, and targeted confirmatory testing, strategies that have been advocated to mitigate overdiagnosis in populations susceptible to asymptomatic bacteriuria [68,69,70,71,92]. This constitutes an approach that balances the technical sensitivity of NAATs with the need to preserve clinical specificity in routine practice. As these design choices are refined and validated against patient-centered outcomes, hybridization-based assays such as CAPTURE UTI possess the potential to complement amplification-based tests by providing a scalable, low-background, and viability-aware option for high-throughput clinical workflows.

NGS-based assays epitomize the most sophisticated frontier in UTI diagnostics, possessing the capacity to concurrently identify myriad bacterial, fungal, viral, and resistance gene targets within a singular assay. In contrast to targeted PCR, NGS affords a holistic perspective of the urinary microbiome, facilitating the recognition of rare, fastidious, or unforeseen pathogens that conventional diagnostics may overlook. Furthermore, sequencing-based methodologies permit the characterization of AMR determinants across an extensive genetic spectrum, bolstering the surveillance of five out of twenty-two resistance trends and informing precision therapeutics [88]. Notwithstanding these advantages, NGS presently encounters substantial practical impediments. Turnaround durations of up to 24 h, combined with the necessity for specialized sequencing platforms and bioinformatics acumen, constrain its applicability to urgent clinical contexts. Financial considerations remain another significant barrier, particularly in low- and middle-income nations where the UTI burden is disproportionately elevated. Nevertheless, as sequencing expenditures diminish and portable sequencers gain broader accessibility, NGS harbors the potential to transition from a research instrument to a clinical reality. Moreover, the capability to amalgamate pathogen detection with host–response biomarkers could revolutionize NGS into a comprehensive diagnostic modality. Consequently, NGS signifies a formidable yet still nascent approach, bridging microbiology, genomics, and clinical medicine [90].

NGS-based assays epitomize the most comprehensive molecular methodology for the diagnostics of urinary tract infections, as they are capable of concurrently detecting bacterial, fungal, viral, and resistance determinants within a singular analytical run; in practice, numerous offerings are presently arranged as reference-laboratory services rather than point-of-care tests [72]. In addition to shotgun methodologies, targeted capture has permeated the domain of urine diagnostics: for instance, Illumina’s urine-specific research-use-only Urinary Pathogen ID/AMR enrichment kit delineates up to 121 bacterial species, 3728 antimicrobial resistance genes, 14 fungi, 4 parasites, and 35 viruses within approximately twenty-four hours [75]. The evidentiary foundation exhibits promise yet also heterogeneity. A contemporary meta-analysis encompassing seven studies with 274 urine specimens revealed that 78.1% of culture-negative specimens were positive via sequencing, and that species diversity was substantially greater through sequencing than through conventional culture methods, with 170 bacterial taxa identified by sequencing in comparison to 38 cultivable via standard procedures; only two studies endeavored in silico antimicrobial susceptibility predictions, which were concordant in 84.6% of 52 specimens [72]. Furthermore, one comparative investigation of symptomatic acute cystitis documented more pronounced enhancements in symptom scores when therapeutic interventions were directed by sequencing rather than by culture (8.5 versus 3.7), yet in an asymptomatic control cohort, five of twenty-two exhibited positive cultures while twenty-one of twenty-two demonstrated positive sequencing outcomes at a mean concentration of 10^5^ CFU/mL, highlighting the peril of over-diagnosis in the absence of meticulous stewardship [76]. A pragmatic solution to this challenge is the integration of sequencing results with clinical context, including the presence of symptoms, urinalysis or pyuria markers, and semi-quantitative reporting thresholds (e.g., ≥10^4^–10^5^ CFU/mL). Such approaches can suppress the reporting of low-level detections in asymptomatic individuals and help distinguish true infection from colonization, thereby mitigating unnecessary antimicrobial therapy. Accordingly, an emerging consensus indicates that sequencing contributes minimally to culture for uncomplicated cystitis, yet it may facilitate the management of complicated or recurrent infections and in conditions such as interstitial cystitis, overactive bladder syndrome, and bladder pain syndrome, where uncultivable organisms may exacerbate symptoms [77,78]. Consequently, practical implementation should underscore semi-quantitative reporting (for instance, colony-forming-unit-equivalent bands), reflex phenotypic susceptibility confirmation for treatment-limiting determinations, and testing algorithms that prioritize symptomatic patients, while acknowledging that turnaround time, platform expenses, and bioinformatics proficiency continue to constrain urgent, extensive clinical application [72,76].

Over the preceding five years, considerable advancements have been accomplished in the advancement of expedited phenotypic antimicrobial susceptibility testing (AST) platforms that are currently being extended from blood cultures to urine specimens [7,93,94,95]. These technologies aim to abbreviate the diagnostic interval by furnishing susceptibility outcomes within 4–8 h, significantly more swiftly than traditional broth microdilution or disk diffusion methodologies. Approaches under scrutiny encompass microfluidic imaging, light-scattering methodologies, impedance cytometry, and nanoparticle-mediated signal amplification [93]. A prevalent characteristic of several of these platforms is their provision of susceptibility outcomes devoid of a definitive organism identification phase, which persists as a contentious matter within the domain [7,94]. While some authorities contend that organism identification may not invariably be imperative for the management of uncomplicated cystitis, regulatory stipulations such as those from the College of American Pathologists necessitate that AST results be associated with a specific organism on the final laboratory report [96]. Consequently, the discourse reflects a dichotomy between expediting decision-making and ensuring diagnostic rigor, underscoring the necessity for outcome-driven investigations that scrutinize the clinical ramifications of such streamlined methodologies.

Among the platforms under vigorous development, nanofluidic systems such as Astrego’s technology assess bacterial proliferation and morphological alterations at the single-cell level, yielding reliable susceptibility outcomes in less than one hour for certain antimicrobials [79,80]. This system has exhibited high sensitivity and specificity for bacteriuria and has already commenced clinical evaluation through a commercial collaboration, emphasizing its translational viability [81]. Similarly, the Alfred 60/AST system, extensively utilized in European laboratories, employs laser light scattering to gauge growth kinetics and has demonstrated strong concordance with standard methodologies, although it has yet to secure FDA endorsement [61]. Other platforms, such as ASTsense utilize nanoparticle-mediated signal amplification, producing colorimetric readouts that correlate with antimicrobial resistance, while the JIDDU system employs microfluidics to evaluate bacterial metabolic activity utilizing fluorescent probes [82]. Collectively, these technologies exemplify the expanding diversity of phenotypic AST strategies, ranging from miniaturized optics to biosensor-based readouts. However, the majority of systems remain in the nascent stages of clinical validation, and their incorporation into practice will hinge upon robust multicenter trials, regulatory approval, and cost-effectiveness evaluations.

Other significant methodologies encompass impedance flow cytometry (iFAST), which assesses electrical and morphological characteristics of bacterial cells to deduce susceptibility, and U-DETECT, which employs an innovative dual-enzyme amplification system to identify extended-spectrum β-lactamases (ESBLs) directly from urine samples [83,84,85]. These techniques are particularly promising for swiftly recognizing resistant phenotypes such as ESBL producers, facilitating clinicians to customize therapy expeditiously and evade inappropriate antimicrobial exposure. Nonetheless, obstacles persist. Numerous platforms concentrate on singular or constrained resistance mechanisms, which may limit their clinical applicability in heterogeneous patient populations [86]. Furthermore, the lack of integrated pathogen identification, substantial development expenditures, and the necessity for laboratory infrastructure may restrict extensive adoption, particularly in low- and middle-income nations where the UTI prevalence is highest. Notably, prospective investigations that evaluate patient outcomes, antibiotic stewardship advantages, and health economic ramifications are urgently warranted. Consequently, while rapid phenotypic AST platforms symbolize a pivotal advancement toward personalized UTI management, their ultimate worth will hinge on demonstrating clinical efficacy, cost-effectiveness, and scalability across healthcare systems.

## 6. Future Perspectives

Future advancements in UTI diagnostics will be significantly contingent on the enhancement of clinical validation through extensive, multicenter trials and subsequent regulatory endorsement procedures. Currently, the majority of innovative molecular platforms remain restricted to investigative or limited clinical applications, with only a small proportion having undergone stringent assessment across varied patient cohorts. Attaining in vitro diagnostic classification necessitates the demonstration of both analytic validity and clinical utility, which must be substantiated under standardized regulatory frameworks. While the technological heterogeneity of nascent assays is encouraging, their widespread integration will depend on evidence that they consistently enhance patient outcomes in comparison to traditional culture-based methodologies. Furthermore, cross-calibration with established culture benchmarks remains imperative to ensure interpretability, particularly when nucleic acid amplification tests identify low colony counts or nonviable organisms. In the absence of such harmonization, there exists a peril of overdiagnosis or unwarranted treatment. Future investigations must therefore not only emphasize sensitivity and specificity but also assess real-world clinical endpoints, such as reductions in hospitalization, antibiotic misuse, and healthcare expenditures. Consequently, broader international regulatory convergence and standardized norms will be vital to expedite the translation of these technologies from promising prototypes into regular clinical practice.

An important limitation is that most innovative molecular diagnostic platforms remain in investigational or restricted clinical use. As a result, their findings should be interpreted with caution, accompanied by appropriate explanatory statements to minimize the risk of misapplication in patient care. This limitation highlights the gap between technological capability and clinical readiness. Therefore, ongoing large-scale validation studies and the development of standardized interpretative frameworks are essential before these methods can be widely implemented in routine diagnostics.

Another significant prospective trajectory pertains to achieving an equilibrium between analytical sensitivity and clinical applicability. As innovative assays become progressively adept at identifying organisms at exceedingly low thresholds, the interpretation of such findings becomes intricate. Molecular methodologies may discern organisms at levels conventionally deemed insignificant or may detect DNA from nonviable or dormant bacteria. This engenders a quandary for clinicians, as not all detected signals possess clinical relevance. Consequently, reporting strategies must advance to prioritize salient results. One potential resolution is the implementation of semi-quantitative or tiered reporting frameworks, which contextualize pathogen load and assist providers in differentiating between colonization and infection. Furthermore, the incorporation of resistance gene detection presents an opportunity to enhance empirical prescribing practices; however, correlating these findings to treatment guidelines remains convoluted when resistance determinants do not align directly with clinical outcomes. Moreover, the execution of such nuanced reporting will necessitate collaboration among diagnostic manufacturers, professional societies, and healthcare payers to delineate thresholds that are both clinically pertinent and economically viable. Ultimately, the future of UTI diagnostics resides in the development of assays that yield not only precise laboratory data but also results that are directly translatable into clinical decision-making, thereby optimizing both patient care and antimicrobial stewardship.

The economic aspect of innovative UTI diagnostics embodies a considerable impediment to widespread implementation and consequently necessitates meticulous attention in the future. Molecular platforms are frequently significantly more costly than culture-based testing, eliciting apprehensions among healthcare systems, payers, and laboratories regarding cost-effectiveness. Reimbursement frameworks have historically constrained coverage for multiplex molecular panels, as evidenced in syndromic testing for respiratory and gastrointestinal pathogens, where such assays are reimbursed solely under limited clinical indications [96]. Consequently, UTI diagnostics must substantiate tangible cost savings through reductions in hospital admissions, diminished emergency visits, and lower rates of inappropriate antibiotic utilization. To realize this, developers will need to undertake rigorous health economic analyses that encompass not only laboratory costs but also downstream clinical advantages. Furthermore, reimbursement models will need to evolve to incentivize the adoption of high-value diagnostics that support stewardship initiatives. Laboratories may benefit from multitarget assays that offer more favorable reimbursement structures; however, cost-benefit analyses must be meticulously tailored to diverse patient populations. As these economic dynamics progress, collaboration among manufacturers, regulators, and insurers will be imperative to ensure that novel diagnostics can be sustainably integrated into healthcare systems. Looking ahead, we anticipate that ongoing cost reductions in sequencing, the advent of portable platforms, and simplified workflows will gradually mitigate the current financial and infrastructural barriers. These innovations, together with evolving reimbursement models, suggest that the future of UTI diagnostics, while economically challenging at present, remains highly promising for scalable clinical integration. Hence, the future of innovation will be dictated as much by financial viability as by technological advancement.

Looking ahead, UTI diagnostics will progressively extend beyond pathogen identification to encompass insights from the urinary microbiome and host-response biomarkers [95]. Investigations have elucidated that urine is not devoid of microorganisms but contains a dynamic microbial consortium, and the clinical relevance of nontraditional organisms remains inadequately comprehended. Molecular assays that identify both classical pathogens and commensal species challenge established definitions of infection versus colonization [96]. Consequently, the assimilation of host inflammatory markers, such as cytokines or neutrophil-associated proteins, could provide contextual differentiation that distinguishes clinically pertinent infection from asymptomatic bacteriuria. Moreover, the amalgamation of pathogen detection with host-response profiling could enhance risk stratification, identifying patients who are more predisposed to develop recurrent or complicated infections. Future diagnostics may therefore embrace a multi-omic methodology that integrates microbial, genetic, and immunological data into a unified platform. Such amalgamation would augment diagnostic precision and assist in guiding personalized therapy; however, it also introduces intricacy in interpretation and prompts inquiries regarding clinical guidelines [97]. Hence, forthcoming endeavors must encompass translational research that substantiates these markers in prospective trials and establishes frameworks for their integration into clinical decision-making, thereby advancing UTI diagnostics toward a more holistic, patient-centered paradigm.

Finally, the prospective success of innovative UTI diagnostics will hinge on seamless incorporation into prevailing clinical workflows. Technologies that provide swift and dependable results are only advantageous if they align with provider decision-making and healthcare infrastructure. For instance, assays that present pooled antimicrobial susceptibility testing may yield intricate reports that necessitate clinician interpretation in the absence of explicit guidelines. Moreover, direct promotion of diagnostics to providers, circumventing institutional oversight, raises ethical and practical dilemmas. Looking forward, diagnostic platforms must be combined with decision-support tools that translate results into actionable recommendations without encroaching upon clinical judgment. Integration with electronic health records and antimicrobial stewardship programs will also be pivotal for optimizing clinical utility. Beyond this, future innovations are likely to investigate artificial intelligence (AI)-driven methodologies that amalgamate laboratory data, patient risk factors, and treatment algorithms to bolster personalized therapy [96,98,99]. Consequently, the trajectory of UTI diagnostics will encompass not only ongoing refinement of molecular techniques but also the development of intelligent, clinician-friendly systems that enhance patient care in a sustainable and evidence-based manner.

## 7. Conclusions

The diagnostic assessment of urinary tract infections has advanced significantly since the mid-20th century; nevertheless, urine culture persists as the benchmark against which novel technologies are evaluated. While culture retains clinical value, its inherent delays, limited sensitivity for low-abundance or fastidious organisms, and inability to reliably distinguish infection from colonization highlight critical gaps in timely and accurate care.

This review underscores that molecular approaches such as NAATs, DNA/RNA hybridization systems, and NGS represent the most promising avenues for bridging these gaps. Their ability to deliver rapid, multiplexed pathogen detection and resistance gene profiling positions them as pivotal tools for improving diagnostic turnaround, particularly in complicated and recurrent UTIs where conventional culture is insufficient. Phenotypic innovations also contribute by accelerating susceptibility testing and creating opportunities for more precise antimicrobial stewardship.

The core contribution of this work is a critical synthesis of how emerging platforms address longstanding diagnostic challenges while also clarifying their limitations, including the risk of overdiagnosis in asymptomatic bacteriuria, interpretive uncertainty in polymicrobial contexts, and unresolved concerns regarding cost-effectiveness. By integrating clinical, regulatory, and economic perspectives, we provide a framework to assess which technologies are ready for near-term implementation and which require further validation.

Looking ahead, successful integration of novel diagnostics into clinical workflows will depend on multicenter validation, harmonization with culture thresholds, and rigorous cost-effectiveness analyses. Most importantly, the next generation of UTI diagnostics must demonstrate not only technical sensitivity but also measurable improvements in patient outcomes, reduced inappropriate antibiotic use, and enhanced management of complicated cases. In this way, the field is evolving from a culture-centric paradigm toward a multifaceted diagnostic model that incorporates molecular and phenotypic innovation, ensuring that advances translate into tangible benefits for patients and healthcare systems.

## Figures and Tables

**Table 1:** Preanalytical limitations, diagnostic flags, and reflex actions aligned with mitigation strategies.

Preanalytical Limitation	Typical Indicator at Accession	Risk to Interpretation	Reflex Action When Suspected	Companion Data to Consider	Expected Effect of Mitigation
Delay to plating at room temperature beyond about 30 min to 2 h	Undocumented transport time or reported delay	Overgrowth of periurethral flora and shift in relative abundance	Repeat collection with reinforced instructions and process immediately or hold at 4 °C; if repeat is not feasible, interpret with caution	Pyuria on microscopy, leukocyte esterase or nitrite on dipstick, symptom severity	Reduces mixed growth and false positives; improves quantitative fidelity [41,42,43,44]
Prolonged cold storage or extended exposure to boric acid preservative	Time in cold storage exceeds local policy or approaches 48 h	Loss of viability for some fastidious organisms and underdetection	Repeat collection and process promptly; consider enhanced quantitative urine culture with larger inoculum and extended incubation	Pyuria and clinical trajectory	Restores recovery of slow-growing or fastidious taxa [38,41,42,43,44,45]
Uncertain midstream clean catch quality	Abundant squamous epithelial cells or visible contamination	Mixed growth that mimics contamination and obscures true pathogen	Repeat collection with coached clean catch; for children, consider catheter or suprapubic aspiration when clinically justified	Pyuria, symptom pattern, fever	Improves purity and clarity of the culture readout [40,42,46,47]
Infant bag collection	Bag method documented	High contamination rate and unreliable quantitation	Prefer catheter or suprapubic aspiration for diagnostic purposes; repeat if feasible	Pyuria and clinical assessment	Lowers contamination and supports reliable interpretation [40]
Menstruation or heavy vaginal discharge at collection	History at accession or visible blood or discharge	Increased mixed growth and misclassification of contamination	Repeat after menstruation when feasible; reinforced clean catch; consider larger inoculum with extended conditions if urgent	Pyuria, nitrite, symptom context	Lowers false positives due to contamination [42,46]
Long catheter dwell or sampling from drainage bag	Sample taken from bag or unknown dwell time	Biofilm-derived organisms and misleading counts	Resample from catheter port after appropriate disinfection or replace catheter prior to collection	Pyuria, systemic signs, device history	Improves specificity for catheter-associated infection [42,46,47]
Recent antibiotic exposure within the previous 24 to 48 h	Medication history positive for recent antibiotics	Suppressed growth despite ongoing symptoms and false negatives	Repeat collection after antibiotic washout when safe; increase inoculated volume and apply enhanced culture conditions	Pyuria, C-reactive protein if available, symptom trajectory	Increases yield when viable organisms are present at low level [35,45]
Low inoculated volume in a patient with high clinical probability	One microliter protocol documented with borderline counts	Counts near the detection limit and missed low-level infection	Increase inoculated volume to ten microliters or more, and consider enhanced quantitative urine culture	Pyuria, nitrite, prior results	Lowers false negatives near the threshold of detection [35,42,46,47]
Mixed growth with three or more morphotypes	Report notes multiple distinct colony types	Assumed contamination may mask true polymicrobial infection in selected hosts	Repeat collection and increase inoculum; if clinical probability is high, apply enrichment media and atmospheres with longer incubation	Pyuria, host risk factors, recent procedures	Distinguishes contamination from clinically meaningful polymicrobial infection [35,47]
Borderline quantitative counts near 10^2^ to 10^3^ CFU per mL with symptoms	Quantitation close to the laboratory limit of detection	Ambiguity between contamination and early infection	Repeat collection, increase inoculum, and use enhanced culture conditions; interpret alongside inflammation and symptoms	Pyuria, leukocyte esterase, nitrite, pain pattern	Clarifies low-level results and reduces both false negatives and false positives [34,35,42,46]

**Table 2:** Scenario-specific interpretation of semi-quantitative urine culture with integrated preanalytical pitfalls and reflex actions.

Clinical Scenario	Collection Method	Preferred Inoculum for Plating	Threshold for High Likelihood of Clinical Significance	High-Risk Preanalytical Pitfalls to Watch	Diagnostic Flags at Accession	Reflex Actions if Borderline or Conflicting	Companion Clinical Data to Weigh	Expected Effect of Mitigation	Notes and References
Adult outpatient without catheter	Midstream clean catch	≥10 µL when clinical probability is high	≥10^5^ CFU per mL for a single uropathogen	Delay to plating at room temperature, uncertain cleansing, menstruation or heavy discharge, very small inoculum	Undocumented transport time, abundant squamous cells, visible blood or discharge	Repeat coached clean catch, process immediately or hold at 4 °C, increase inoculum, consider enhanced culture if symptoms persist	Pyuria, nitrite, symptom severity and trajectory	Reduces mixed growth and false positives, improves detection near the limit	Storage/preservative practices [36,37,38,39]; thresholds and mixed growth interpretation [34,42]; enhanced culture utility [51,52,53,54]
Pregnancy	Midstream clean catch	≥10 µL	≥10^5^ CFU per mL; lower tolerance for mixed growth	Same as adult outpatient; added emphasis on prompt processing	History of delay or suboptimal collection	Repeat promptly, increase inoculum, consider enhanced culture when symptoms persist	Pyuria and clinical assessment	Minimizes contamination and supports timely decisions	Thresholds/collection [34,42]; preanalytical control [36,37,38,39]
Pediatric outpatient	Clean catch if feasible; catheter or suprapubic for diagnosis	≥10 µL for clean catch; standard loop for catheter or suprapubic	Clean catch ≥10^5^ CFU per mL; catheter ≥10^4^ CFU per mL	Bag collection, delay to plating, small inoculum	Bag method documented, abundant squamous cells	Prefer catheter or suprapubic aspiration for diagnosis; repeat if contamination suspected	Pyuria, fever, systemic signs	Lowers contamination and improves quantitative fidelity	Pediatric sampling and thresholds [40,42,46]
Infant diagnostic sampling	Catheter or suprapubic aspiration	Laboratory standard	Catheter ≥10^4^ CFU per mL; any growth from suprapubic aspirate is significant	Any use of bag collection; transport delays	Bag collection recorded; long transport time	Replace bag with catheter or suprapubic aspiration; process immediately or hold at 4 °C	Pyuria and clinical assessment	Improves specificity and reduces false positives	Collection method impact [40,42,46]
Indwelling catheter in place	From catheter port, not drainage bag	Laboratory standard; increase to ≥10 µL if borderline	≥10^4^ CFU per mL for a single uropathogen	Sampling from drainage bag; long catheter dwell time	Sample source recorded as bag; unknown dwell time	Resample from disinfected port or after catheter change; consider enhanced culture	Pyuria, device history, systemic signs	Better discrimination of catheter-associated infection	Catheter sampling and biofilm issues [40,42,46]
After catheter replacement or suprapubic aspiration	Sterile port after replacement or suprapubic aspirate	Laboratory standard	Catheter ≥10^4^ CFU per mL; suprapubic any significant growth	Prolonged cold storage beyond policy; preservative exposure near 48 h	Time in cold storage approaching 48 h	Process promptly; if delayed, repeat collection; consider enhanced culture	Pyuria, pain, fever	Restores recovery of fastidious organisms	Storage/preservative effects [41,42,43,44]; enhanced culture [44,45,46]
Neurogenic bladder or urinary diversion	As appropriate for device and anatomy	≥10 µL if borderline	Often interpret ≥10^4^ CFU per mL with greater weight on symptoms and inflammation	Inadequate cleansing; device colonization; transport delays	Mixed flora with multiple morphotypes	Repeat collection; increase inoculum; apply enhanced media and atmospheres with longer incubation	Pyuria, residual volume, device history	Clarifies mixed growth and detects low-level infection	Host context and polymicrobial risk [46,47,51,52,53]
Immunosuppressed or transplant	Method tailored to clinical status	≥10 µL if borderline	≥10^4^ CFU per mL may be meaningful with symptoms or inflammation	Recent antibiotics; prolonged storage; small inoculum	Recent antimicrobial exposure recorded	Repeat after brief washout when safe; increase inoculum; apply enhanced culture	Pyuria, systemic markers, symptom course	Increases yield when viable organisms are present at low level	Low-count symptomatic infection [44,45,46,47]; antibiotic impact [42,50]
Recent antibiotics within 24–48 h	Any method with exposure documented	≥10 µL	Numeric thresholds are less reliable due to suppressed growth	Antibiotic exposure; transport delay	Medication history positive for recent antibiotics	Repeat after washout when safe; increase inoculum; use enhanced culture	Pyuria, C-reactive protein if available, symptom trajectory	Restores detection near the limit and reduces false negatives	Antibiotic suppression and reflex strategy [46,47,51,52,53]
Recurrent symptoms with prior negative culture	Same or upgraded collection method	≥10 µL	Treat any quantitative result with caution; pattern over time matters	Any of the above depending on setting	Prior negative culture with high clinical probability	Repeat with larger inoculum and enhanced culture; consider molecular profiling if available; integrate symptoms and inflammation	Pyuria, prior results, analgesic or antibiotic use	Clarifies discordant results and guides targeted work-up	Reflex rules and enhanced detection [46,47,51,52,53]
Mixed growth reported	Three or more distinct morphotypes or mixed flora	≥10 µL on repeat	Often contamination in low-risk settings; can be polymicrobial disease in selected hosts	Uncertain clean catch quality; menstruation; device colonization	Abundant squamous cells; mixed colony types	Repeat coached clean catch; increase inoculum; in high-risk hosts apply additional media, alternative atmospheres, and longer incubation	Pyuria, host risk factors, procedure history	Distinguishes contamination from clinically meaningful polymicrobial infection	Mixed growth interpretation and enhanced culture [46,47,51,52,53]

**Table 3:** Innovative diagnostic methods for UTIs and their clinical relevance.

Method	Detection Principle	Typical Time to Result	Strengths	Limitations	Best-Fit Clinical Use	Example Platforms and Notes	References
Bacteriuria screening assays	Surrogate markers of infection or non-specific bacterial detection by light scattering, enzyme activity, or electrochemical signals	Minutes to ~3 h	Very high negative predictive value; reduces unnecessary cultures; can fast-track downstream testing	Positive predictive value for infection can be low; does not identify the organism	Triage of routine specimens; early rule-out; selection of samples for direct mass-spectrometry identification	BacterioScan 216Dx (~3 h; sensitivity ~96.5%, specificity ~72%; cleared by the United States Food and Drug Administration); Alifax Uro-Quick (~3 h; available outside the United States); Usense Jimini (~5 min; host inflammatory and bacterial targets); Uriscreen (catalase activity; modest performance in pregnancy and pediatrics); UTRIPlex (three urinary inflammatory markers; high specificity, low sensitivity in pediatrics); UTI-lizer (microreactor “digital dipstick,” ~8 h; very high negative predictive value, widely variable positive predictive value depending on organism); Pattern single-cell platform (negative predictive value ~98% for bacteriuria and promising Gram classification)	[58,59,60,61,62,63,64,65,66,67]
Rapid molecular identification (multiplex nucleic acid amplification)	Direct amplification of microbial nucleic acids from urine; multiple targets per panel	Hours	Rapid; high analytical sensitivity; can detect difficult-to-culture organisms	May detect nucleic acids from nonviable organisms after recent antibiotics; target lists are finite; interpretation requires clinical correlation when inflammation is absent or signals are low	High clinical probability with negative or borderline culture; recent antibiotics; situations where early organism-directed therapy could change outcomes	Commercial multiplex panels; reports should state target coverage and unresolved gaps	[68,69,70,71,72]
Next-generation sequencing	Community profiling by sequencing (for example, 16S ribosomal RNA gene sequencing or shotgun sequencing)	~1–3 days (laboratory dependent)	Detects difficult-to-grow and non-culturable organisms; useful in complicated or recurrent urinary infections	May detect commensals in people with and without symptoms; interpretation is complex; does not provide phenotypic susceptibility	Complex or recurrent presentations; discordant culture findings; targeted discovery rather than routine frontline testing	Requires standardized workflows and outcome-linked reporting; integrate with symptoms, inflammation, and collection method	[71,72,73,74,75,76,77,78]
Rapid phenotypic antimicrobial susceptibility testing	Measures bacterial growth or metabolic activity in the presence of antibiotics directly in urine	Hours	Rapid evaluation of antimicrobial susceptibility; potential to move from empirical to targeted treatment on the same day	Requires sufficient bacterial burden; scope of antibiotic panels varies; usually needs accompanying culture for organism identification and linkage of result	High-probability cases where early escalation or de-escalation could change outcomes	Pattern Biosciences single-cell droplet imaging under development; additional optical and electrochemical platforms are emerging	[79,80,81,82,83,84,85]

**Table 4:** Modern urine bacteriuria screening tools: principle, speed, clinical role, and practical considerations.

Test and Platform (Manufacturer)	Regulatory Status	Detection Principle	Clinical Role This Test Answers	Time to Result	Specimen Handling and Throughput	Stated Limit of Detection (Colony-Forming Units per Milliliter)	Suitable for Near-Patient Use	Can Feed Same-Day Matrix-Assisted Laser Desorption Ionization Time-of-Flight Mass Spectrometry?	Reported Performance Highlights	Notes and Caveats	References
UTI-lizer Digital Dipstick (Utilizer, Quotient Diagnostics Ltd., Camberley, UK)	Research use only	Miniaturized culturing in sealed microreactors with species-linked color change	Point-of-care mini-culture that estimates bacterial load and triages need for full culture	About 8 h	Single specimen	About 253 colony-forming units per milliliter	Yes	No	Negative predictive value above 97 percent; positive predictive value varies widely by organism from 0 to 90 percent	Requires short incubation; visual readout demands training and quality control	[65]
Urine screen (Pattern Biosciences, Austin, TX, USA)	Research use only	Single-cell compartmentalization in droplets with fluorescence imaging and metabolic profiling	Rapid bacteriuria detection and organism class prediction; platform under development for direct susceptibility testing	About 2 h	Single specimen	Not applicable	Yes	Potentially, depending on biomass and workflow	Negative predictive value near 98 percent; promising accuracy for Gram classification	Development pathway includes direct organism identification and rapid phenotypic susceptibility testing	[66]
216Dx UTI System (BacterioScan, Inc., St. Louis, MI, USA)	Cleared as an in vitro diagnostic device by the United States Food and Drug Administration	Laser light scattering to quantify bacterial biomass	Rapid rule-out or rule-in of bacteriuria and triage to culture or downstream testing	About 3 h	Urine; up to about 20 specimens per day	About 50,000 colony-forming units per milliliter	No	Yes, when biomass is sufficient	Sensitivity about 96.5 percent; specificity about 72 percent versus culture	Does not identify organism; positive screens still need organism identification and susceptibility testing	[58,59,60,61]
Rapid Urine Culture Test (Alifax S.r.l., Padua, Italy)	Conformité Européenne in vitro diagnostic marking	Light scattering with dedicated analyzers	Same as above; also used to gate direct mass-spectrometry identification	Less than 4 h	Urine; about 60 to 420 specimens depending on analyzer	About 30,000 colony-forming units per milliliter	No	Yes	Widely adopted outside the United States; performance depends on instrument and workflow	Requires analyzer platform (for example, Alfred or HB&L)	[60,61]
Uriscreen (Savyon Diagnostics Ltd., Ashdod, Israel)	Conformité Européenne in vitro diagnostic marking	Detection of catalase activity as a proxy for bacteria in somatic cells	Very rapid triage where culture capacity is limited	About 2 min	Single specimen	Not applicable	Yes	No	Pregnancy study: sensitivity 60.7 percent, specificity 89.3 percent; pediatric ambulatory study: sensitivity 67 percent, specificity 69 percent	Modest accuracy relative to culture; best used as a fast rule-out when prevalence is low	[63,64]
UTRIPlex (Global Access Diagnostics, Thurleigh, Bedfordshire, UK)	Research use only	Lateral-flow detection of three urinary inflammatory markers, including matrix metalloproteinase-8 and human neutrophil elastase	Host-response screen to prioritize culture in children	About 6 min	Single specimen	Not applicable	Yes	No	In pediatrics: sensitivity 21 percent and specificity 94 percent	High specificity but low sensitivity; useful only as a rule-in when positive	[64]
Automated urinalysis analyzers (flow cytometry or digital microscopy, (e.g., Sysmex Corporation, Kobe, Japan; Beckman Coulter, Brea, CA, USA; Siemens Healthineers, Erlangen, Germany)	Varies by model; cleared as in vitro diagnostic devices in many regions	Automated counting of bacteria and leukocytes and imaging of urinary sediment	High-throughput screening and triage with programmable thresholds; supports early rule-out of bacteriuria	About 2 min	Urine; high throughput with continuous loading depending on analyzer	Instrument-specific; typically reported as counts per microliter rather than colony-forming units per milliliter	No	No	High negative predictive value when thresholds are optimized; enables laboratory-scale triage before culture	Requires benchtop analyzer, calibration, and local validation of thresholds; does not identify organism	[67]

**Table 5:** Comparative matrix of molecular urine tests [85,86,87,88,89,90,91,92].

Test and Platform (Manufacturer)	Regulatory Status	Method (Spelled Out)	Targets Reported (Identification and Resistance)	Approximate Time to Result	Specimen and Throughput	Analytical Sensitivity (Limit of Detection)	Platform Requirements and Near-Patient Suitability	Distinctive Notes
Nucleic acid amplification assays (NAATs)								
CLONeT *E. coli* Assay for urinary infection	Research use only	Multiplex polymerase chain reaction with lateral-flow readout	Organism identification: *E. coli*; resistance genes: none reported	Less than 45 min	Urine; one specimen per run	About 100 to 1000 CFU/mL	Multiple real-time thermocyclers; suitable for near-patient use	Narrow organism coverage with very rapid time to result
Usense module (Module Innovations Pvt. Ltd., Pune, India)	Research use only	Signal amplification by nanoparticles (size-dependent)	Organism identification: not disclosed; resistance genes: none reported	About 15 min	Urine; one specimen per run	No data	Designed for near-patient use	Very rapid screen; target list not disclosed in provided source
Lodestar urinary infection test (Llusern Scientific Ltd., Cardiff, UK)	United Kingdom Conformity Assessment	Loop-mediated amplification	Organism identification: six targets; resistance genes: none reported	About 30 min	Urine; one specimen per run	About 10,000 CFU/mL	Llusen Lodestar DX analyzer; suitable for near-patient use	Short turnaround with dedicated analyzer
Randox urinary infection panel (Randox Laboratories Ltd., Crumlin, Northern Ireland, UK)	Research use only	Multiplex real-time polymerase chain reaction	Organism identification: twenty-three targets, including one *Candida* species; resistance genes: eight targets	Less than 4 h	Urine; one specimen per run	About 10,000 CFU/mL	Vivalytic platform; designed for near-patient use	Broad panel for organism identification and resistance markers
PathoKey MP urinary infection identification and antimicrobial resistance polymerase chain reaction test (Vela Diagnostics Pte. Ltd., Singapore (headquarters); also with operations in Hamburg, Germany)	Research use only	Multiplex real-time polymerase chain reaction	Organism identification: thirteen targets, including one *Candida* species; resistance genes: fourteen targets	At least 4 h	About forty samples per run	About 1000 CFU/mL	Multiple real-time thermocyclers; not designed for near-patient use	Combined organism and resistance panel
OpenArray urinary tract microbiota (Thermo Fisher Scientific, Waltham, MA, USA)	Research use only	Multiplex polymerase chain reaction on OpenArray plates	Organism identification: sixteen bacterial targets and one *Candida* species; resistance genes: none reported	About 5 h	Forty-eight to one hundred ninety-two specimens per run	No data	QuantStudio 12K Flex with OpenArray block; not designed for near-patient use	High-throughput research-focused panel
Multiple primer sets targeting urinary infection pathogens (BioGX, Inc., Birmingham, AL, USA)	Research use only	Multiplex real-time polymerase chain reaction	Organism identification: forty-two bacterial and eight fungal targets; resistance genes: eleven targets	About 2 h	Twenty-four to ninety-six specimens per run; urine neat or preserved in boric acid	No data	Platforms include BD Max, ABI, and Bio-Rad; not designed for near-patient use	Large multi-organism panel for laboratory analyzers
QIAstat-Dx complicated urinary infection plus antimicrobial resistance panel (Qiagen N.V., Hilden, Germany)	Research use only	Multiplex real-time polymerase chain reaction	Organism identification: no data; resistance genes: no data in source	Day-scale throughput about 160 specimens	System-integrated cartridge platform	No data	QIAstat-Dx instrument; not designed for near-patient use	Integrated cartridge analyzer; details not provided in source
Unyvero urinary infection panel (Curetis GmbH (a subsidiary of OpGen, Inc.), Bodelshausen, Germany)	Research use only	Multiplex polymerase chain reaction	Organism identification: twenty-three bacterial targets and four *Candida* species; resistance genes: fifteen targets	About 5 h	Two to twelve specimens per run; midstream, suprapubic, or fresh catheter specimens	About 4000 to 100,000 CFU/mL	Unyvero system; not designed for near-patient use	Broad panel with flexible specimen types
DNA/RNA hybridization								
CAPTURE UTI (GeneCapture, Inc., Huntsville, AL, USA)	Research use only (prototype referenced)	Unamplified ribonucleic acid expression detected by probe capture	Organism identification: six bacterial targets and one *Candida* species; resistance genes: none reported	About 2 h	Urine; approximately forty specimens per run	About 10,000 CFU/mL	GeneCapture benchtop instrument; not designed for near-patient use	Representative capture-hybridization approach among urine platforms in development in provided sources
Targeted NGS								
Urinary Pathogen Identification and Antimicrobial Resistance Enrichment Kit (Illumina, Inc., San Diego, CA, USA)	Research use only	Targeted deoxyribonucleic acid sequencing with enrichment	Organism identification: one hundred twenty-one bacterial targets, fourteen fungal, four parasites, and thirty-five viruses; resistance genes: three thousand seven hundred twenty-eight targets	About 1 day	Urine; up to 384 specimens per run (instrument-dependent).	Demonstrated to about 100,000 CFU/mL	Illumina next-generation sequencing instruments; not designed for near-patient use	Very broad detection with extensive resistance catalog; requires sequencing infrastructure

## Data Availability

Not applicable.
